# Crystal structure of *N*-(3-chloro-1-methyl-1*H*-indazol-5-yl)-4-meth­oxy­benzene­sulfonamide

**DOI:** 10.1107/S1600536814017747

**Published:** 2014-08-09

**Authors:** Hakima Chicha, El Mostapha Rakib, Ahmed Gamouh, Mohamed Saadi, Lahcen El Ammari

**Affiliations:** a Laboratoire de Chimie Organique et Analytique, Université Sultan Moulay Slimane, Faculté des Sciences et Techniques, Béni-Mellal, BP 523, Morocco; b Laboratoire de Chimie du Solide Appliquée, Faculté des Sciences, Université Mohammed V-Agdal, Avenue Ibn Battouta, BP. 1014, Rabat, Morocco

**Keywords:** crystal structure, benzene­sulfonamides, hydrogen bonding

## Abstract

In the title compound, C_15_H_14_ClN_3_O_3_S, the dihedral angle between the planes of the indazole ring system (r.m.s. deviation = 0.007 Å) and the benzene ring is 89.05 (7)°. The meth­oxy C atom deviates from its attached ring by 0.196 (3) Å. In the crystal, inversion dimers linked by pairs of N—H⋯O hydrogen bonds generate *R*_2_^2^(8) loops. The dimers are connected into [010] chains by C—H⋯O inter­actions.

## Related literature   

For the biological activity of sulfonamides, see: El-Sayed *et al.* (2011 ▶); Mustafa *et al.* (2012 ▶); Scozzafava *et al.* (2003 ▶). For similar compounds, see: Abbassi *et al.* (2012 ▶, 2013 ▶); Chicha *et al.* (2014 ▶).
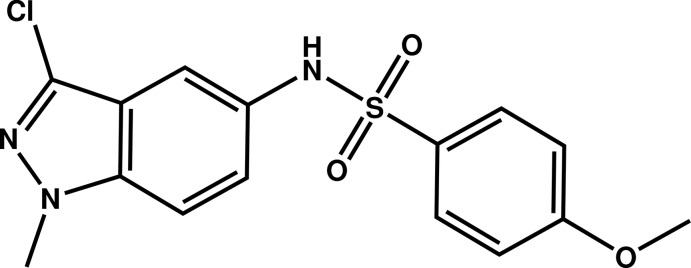


## Experimental   

### Crystal data  

C_15_H_14_ClN_3_O_3_S
*M* *_r_* = 351.80
Triclinic, 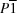

*a* = 8.2023 (1) Å
*b* = 10.6312 (2) Å
*c* = 10.8957 (2) Å
α = 117.523 (1)°
β = 93.095 (1)°
γ = 103.166 (1)°
*V* = 806.36 (2) Å^3^
*Z* = 2
Mo *K*α radiation
μ = 0.38 mm^−1^
*T* = 296 K
0.40 × 0.36 × 0.31 mm

### Data collection  

Bruker X8 APEX CCD diffractometer
Absorption correction: multi-scan (*SADABS*; Bruker, 2009 ▶) *T*_min_ = 0.693, *T*_max_ = 0.747
20115 measured reflections
4526 independent reflections
3707 reflections with *I* > 2σ(*I*)
*R*_int_ = 0.028

### Refinement  

*R*[*F* ^2^ > 2σ(*F* ^2^)] = 0.038
*wR*(*F* ^2^) = 0.116
*S* = 1.03
4526 reflections
209 parameters
H-atom parameters constrained
Δρ_max_ = 0.32 e Å^−3^
Δρ_min_ = −0.37 e Å^−3^

### 

Data collection: *APEX2* (Bruker, 2009 ▶); cell refinement: *SAINT* (Bruker, 2009 ▶); data reduction: *SAINT*; program(s) used to solve structure: *SHELXS97* (Sheldrick, 2008 ▶); program(s) used to refine structure: *SHELXL97* (Sheldrick, 2008 ▶); molecular graphics: *ORTEP-3 for Windows* (Farrugia, 2012 ▶); software used to prepare material for publication: *PLATON* (Spek, 2009 ▶) and *publCIF* (Westrip, 2010 ▶).

## Supplementary Material

Crystal structure: contains datablock(s) I. DOI: 10.1107/S1600536814017747/hb7261sup1.cif

Structure factors: contains datablock(s) I. DOI: 10.1107/S1600536814017747/hb7261Isup2.hkl

Supporting information file. DOI: 10.1107/S1600536814017747/hb7261Isup3.cml

**Fig. 1.**
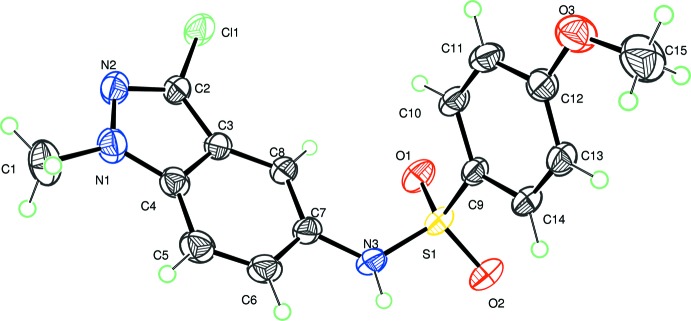
Molecular structure of the title compound with displacement ellipsoids drawn at the 50% probability level.

. DOI: 10.1107/S1600536814017747/hb7261fig1.tif
Mol­ecular structure of the title compound with displacement ellipsoids drawn at the 50% probability level.

**Fig. 2.**
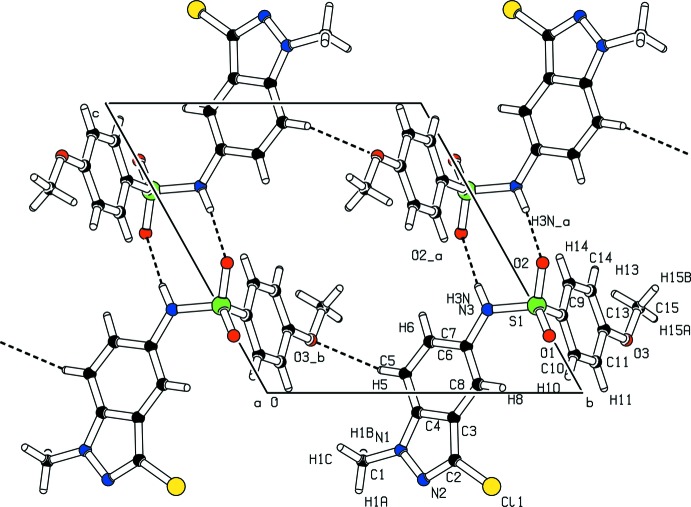
Crystal structure of the title compound, showing molecules linked by N3–H3N···O2 hydrogen bond and forming dimers linked by C5–H5···O3 interaction.

N . DOI: 10.1107/S1600536814017747/hb7261fig2.tif
Crystal structure of the title compound, showing mol­ecules linked by N3–H3*N*⋯O2 hydrogen bond and forming dimers linked by C5–H5⋯O3 inter­action.

CCDC reference: 1017531

Additional supporting information:  crystallographic information; 3D view; checkCIF report

## Figures and Tables

**Table 1:** Hydrogen-bond geometry (Å, °)

*D*—H⋯*A*	*D*—H	H⋯*A*	*D*⋯*A*	*D*—H⋯*A*
N3—H3*N*⋯O2^i^	0.89	2.04	2.9286 (17)	175
C5—H5⋯O3^ii^	0.93	2.56	3.426 (2)	156

Symmetry codes: (i) 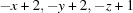; (ii) 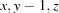.

## supplementary crystallographic information

### S1. Comment

The sulfonamide functional group is a structure with broad importance, as it is found in numerous medicinal agents (El-Sayed, *et al.*, 2011; Mustafa, *et al.*, 2012; Scozzafava, *et al.*, 2003. Previously, we identified a series of indazoles bearing a sulfonamide moiety with good antiproliferative activities (Abbassi, *et al.*, 2012; Abbassi, *et al.*, 2013; Chicha, *et al.*, 2014).

The molecule of the title compound is built up from two fused five- and six-membered rings (N1 N2 C2 to C8) almost coplanar, with a maximum deviation of 0.010 (2) Å for C5 atom (Fig.1). The dihedral angle between the indazol system and the plane through the benzene ring (C9 to C14) is of 89.05 (7)°.

The cohesion of the crystal structure is ensured by N3–H3N···O2 hydrogen bonds between molecules forming dimers, which are linked together by C5–H5···O3 interaction and forming [010] chains as shown in Fig.2 and Table 1.

### S2. Experimental

A mixture of 1-methyl-3-chloro-5-nitroindazole (1.22 mmol) and anhydrous SnCl2 (1.1 g, 6.1 mmol) in 25 mL of absolute ethanol was heated at 60 °C for 6 h. After reduction, the starting material disappeared, and the solution was allowed to cool down. The pH was made slightly basic (pH 7–8) by addition of 5% aqueous potassium bicarbonate before extraction with ethyl acetate. The organic phase was washed with brine and dried over magnesium sulfate. The solvent was removed to afford the amine, which was immediately dissolved in pyridine (5 ml) and then reacted with 4-methoxybenzenesulfonyl chloride (1.25 mmol) at room temperature for 24 h. After the reaction mixture was concentrated *in vacuo*, the resulting residue was purified by flash chromatography (eluted with Ethyl acetate: Hexane 2:8). The title compound was recrystallized from ethanol solution. Yield: 56%, PF: 140–142°C.

### S3. Refinement

H atoms were located in a difference map and treated as riding with C–H = 0.96 Å, C–H = 0.93 Å, and N–H = 0.86 Å for methyl, aromatic CH and NH, respectively. All hydrogen with *U*_iso_(H) = 1.2 *U*_eq_ (aromatic, NH) and *U*_iso_(H) = 1.5 *U*_eq_ for methyl.

### Crystal data

**Table:**

C_15_H_14_ClN_3_O_3_S	*Z* = 2
*M**_r_* = 351.80	*F*(000) = 364
Triclinic, *P*1	*D*_x_ = 1.449 Mg m^−^^3^
Hall symbol: -P 1	Mo *K*α radiation, λ = 0.71073 Å
*a* = 8.2023 (1) Å	Cell parameters from 4526 reflections
*b* = 10.6312 (2) Å	θ = 2.6–29.6°
*c* = 10.8957 (2) Å	µ = 0.38 mm^−^^1^
α = 117.523 (1)°	*T* = 296 K
β = 93.095 (1)°	Block, colourless
γ = 103.166 (1)°	0.40 × 0.36 × 0.31 mm
*V* = 806.36 (2) Å^3^	

### Data collection

**Table:**

Bruker X8 APEX CCD diffractometer	4526 independent reflections
Radiation source: fine-focus sealed tube	3707 reflections with *I* > 2σ(*I*)
Graphite monochromator	*R*_int_ = 0.028
φ and ω scans	θ_max_ = 29.6°, θ_min_ = 2.6°
Absorption correction: multi-scan (*SADABS*; Bruker, 2009)	*h* = −11→11
*T*_min_ = 0.693, *T*_max_ = 0.747	*k* = −14→14
20115 measured reflections	*l* = −15→15

### Refinement

**Table:**

Refinement on *F*^2^	Primary atom site location: structure-invariant direct methods
Least-squares matrix: full	Secondary atom site location: difference Fourier map
*R*[*F*^2^ > 2σ(*F*^2^)] = 0.038	Hydrogen site location: inferred from neighbouring sites
*wR*(*F*^2^) = 0.116	H-atom parameters constrained
*S* = 1.03	*w* = 1/[σ^2^(*F*_o_^2^) + (0.062*P*)^2^ + 0.1878*P*] where *P* = (*F*_o_^2^ + 2*F*_c_^2^)/3
4526 reflections	(Δ/σ)_max_ = 0.001
209 parameters	Δρ_max_ = 0.32 e Å^−^^3^
0 restraints	Δρ_min_ = −0.37 e Å^−^^3^

### Special details

**Table:**

Geometry. All e.s.d.'s (except the e.s.d. in the dihedral angle between two l.s. planes) are estimated using the full covariance matrix. The cell e.s.d.'s are taken into account individually in the estimation of e.s.d.'s in distances, angles and torsion angles; correlations between e.s.d.'s in cell parameters are only used when they are defined by crystal symmetry. An approximate (isotropic) treatment of cell e.s.d.'s is used for estimating e.s.d.'s involving l.s. planes.
Refinement. Refinement of *F*^2^ against all reflections. The weighted *R*-factor *wR* and goodness of fit *S* are based on *F*^2^, conventional *R*-factors *R* are based on *F*, with *F* set to zero for negative *F*^2^. The threshold expression of *F*^2^ > σ(*F*^2^) is used only for calculating *R*-factors(gt) *etc*. and is not relevant to the choice of reflections for refinement. *R*-factors based on *F*^2^ are statistically about twice as large as those based on *F*, and *R*- factors based on all data will be even larger.

### Fractional atomic coordinates and isotropic or equivalent isotropic displacement parameters (Å^2^)

**Table:**

	*x*	*y*	*z*	*U*_iso_*/*U*_eq_	
C1	0.5360 (3)	0.1820 (2)	−0.2354 (2)	0.0641 (5)	
H1A	0.5166	0.1185	−0.3355	0.096*	
H1B	0.4305	0.1986	−0.2072	0.096*	
H1C	0.5801	0.1361	−0.1885	0.096*	
C2	0.8383 (2)	0.47591 (16)	−0.23208 (15)	0.0391 (3)	
C3	0.84060 (18)	0.54753 (16)	−0.08554 (14)	0.0352 (3)	
C4	0.72093 (19)	0.44264 (17)	−0.06796 (16)	0.0397 (3)	
C5	0.6874 (2)	0.4716 (2)	0.06598 (18)	0.0493 (4)	
H5	0.6097	0.4017	0.0787	0.059*	
C6	0.7734 (2)	0.60633 (19)	0.17665 (17)	0.0476 (4)	
H6	0.7515	0.6289	0.2662	0.057*	
C7	0.8951 (2)	0.71369 (17)	0.16058 (14)	0.0385 (3)	
C8	0.93096 (19)	0.68456 (16)	0.02938 (14)	0.0375 (3)	
H8	1.0120	0.7532	0.0177	0.045*	
C9	0.88896 (18)	1.06974 (16)	0.26748 (13)	0.0364 (3)	
C10	0.83288 (19)	1.04012 (17)	0.13088 (14)	0.0407 (3)	
H10	0.8815	0.9839	0.0568	0.049*	
C11	0.7053 (2)	1.09469 (18)	0.10689 (15)	0.0441 (3)	
H11	0.6677	1.0754	0.0162	0.053*	
C12	0.6321 (2)	1.17864 (17)	0.21742 (16)	0.0413 (3)	
C13	0.6836 (2)	1.20357 (17)	0.35215 (16)	0.0435 (3)	
H13	0.6319	1.2566	0.4254	0.052*	
C14	0.8118 (2)	1.14935 (17)	0.37674 (14)	0.0413 (3)	
H14	0.8469	1.1661	0.4670	0.050*	
C15	0.4427 (3)	1.3276 (3)	0.2942 (3)	0.0797 (7)	
H15A	0.3597	1.3573	0.2564	0.120*	
H15B	0.5324	1.4136	0.3600	0.120*	
H15C	0.3895	1.2767	0.3414	0.120*	
N1	0.65760 (18)	0.32210 (15)	−0.19782 (15)	0.0478 (3)	
N2	0.72912 (18)	0.34244 (15)	−0.29918 (14)	0.0456 (3)	
N3	0.98123 (19)	0.84656 (15)	0.28741 (13)	0.0459 (3)	
H3N	0.9455	0.8563	0.3661	0.057 (5)*	
O1	1.16088 (15)	0.99481 (14)	0.19715 (12)	0.0520 (3)	
O2	1.13503 (15)	1.10282 (14)	0.44851 (11)	0.0535 (3)	
O3	0.51174 (17)	1.23162 (16)	0.18293 (14)	0.0575 (3)	
S1	1.05777 (5)	1.00891 (4)	0.30176 (3)	0.04069 (12)	
Cl1	0.95605 (6)	0.54559 (5)	−0.32303 (4)	0.05614 (14)	

### Atomic displacement parameters (Å^2^)

**Table:**

	*U*^11^	*U*^22^	*U*^33^	*U*^12^	*U*^13^	*U*^23^
C1	0.0617 (11)	0.0421 (9)	0.0786 (13)	0.0032 (8)	0.0160 (10)	0.0262 (9)
C2	0.0482 (8)	0.0355 (7)	0.0327 (6)	0.0140 (6)	0.0112 (6)	0.0147 (6)
C3	0.0424 (7)	0.0354 (7)	0.0325 (6)	0.0176 (6)	0.0115 (5)	0.0170 (5)
C4	0.0443 (7)	0.0386 (8)	0.0420 (7)	0.0166 (6)	0.0133 (6)	0.0215 (6)
C5	0.0585 (10)	0.0506 (9)	0.0507 (9)	0.0187 (8)	0.0230 (7)	0.0315 (8)
C6	0.0631 (10)	0.0545 (10)	0.0396 (7)	0.0273 (8)	0.0224 (7)	0.0285 (7)
C7	0.0492 (8)	0.0416 (8)	0.0314 (6)	0.0243 (6)	0.0126 (6)	0.0174 (6)
C8	0.0468 (7)	0.0363 (7)	0.0308 (6)	0.0157 (6)	0.0108 (5)	0.0154 (5)
C9	0.0443 (7)	0.0328 (7)	0.0264 (6)	0.0081 (5)	0.0090 (5)	0.0110 (5)
C10	0.0462 (8)	0.0430 (8)	0.0263 (6)	0.0092 (6)	0.0109 (5)	0.0128 (6)
C11	0.0498 (8)	0.0489 (9)	0.0311 (6)	0.0088 (7)	0.0072 (6)	0.0197 (6)
C12	0.0446 (8)	0.0372 (7)	0.0413 (7)	0.0073 (6)	0.0085 (6)	0.0203 (6)
C13	0.0540 (9)	0.0394 (8)	0.0344 (7)	0.0165 (7)	0.0153 (6)	0.0137 (6)
C14	0.0535 (8)	0.0406 (8)	0.0256 (6)	0.0146 (6)	0.0103 (6)	0.0120 (5)
C15	0.0883 (16)	0.0877 (17)	0.0909 (17)	0.0560 (14)	0.0333 (14)	0.0502 (14)
N1	0.0523 (8)	0.0374 (7)	0.0485 (7)	0.0079 (6)	0.0134 (6)	0.0187 (6)
N2	0.0536 (8)	0.0367 (7)	0.0393 (6)	0.0111 (6)	0.0103 (6)	0.0134 (5)
N3	0.0649 (8)	0.0476 (8)	0.0272 (5)	0.0231 (6)	0.0116 (5)	0.0165 (5)
O1	0.0485 (6)	0.0604 (8)	0.0370 (5)	0.0149 (5)	0.0163 (5)	0.0151 (5)
O2	0.0533 (7)	0.0610 (8)	0.0281 (5)	0.0122 (6)	0.0018 (4)	0.0097 (5)
O3	0.0610 (7)	0.0654 (8)	0.0594 (7)	0.0281 (6)	0.0141 (6)	0.0362 (7)
S1	0.0437 (2)	0.0454 (2)	0.02480 (16)	0.01330 (15)	0.00761 (13)	0.01019 (14)
Cl1	0.0743 (3)	0.0487 (2)	0.0361 (2)	0.0060 (2)	0.01902 (18)	0.01758 (17)

### Geometric parameters (Å, º)

**Table:**

C1—N1	1.448 (2)	C9—S1	1.7465 (16)
C1—H1A	0.9600	C10—C11	1.374 (2)
C1—H1B	0.9600	C10—H10	0.9300
C1—H1C	0.9600	C11—C12	1.392 (2)
C2—N2	1.320 (2)	C11—H11	0.9300
C2—C3	1.4129 (18)	C12—O3	1.357 (2)
C2—Cl1	1.7085 (15)	C12—C13	1.388 (2)
C3—C8	1.401 (2)	C13—C14	1.378 (2)
C3—C4	1.401 (2)	C13—H13	0.9300
C4—N1	1.362 (2)	C14—H14	0.9300
C4—C5	1.403 (2)	C15—O3	1.423 (3)
C5—C6	1.362 (2)	C15—H15A	0.9600
C5—H5	0.9300	C15—H15B	0.9600
C6—C7	1.419 (2)	C15—H15C	0.9600
C6—H6	0.9300	N1—N2	1.3577 (19)
C7—C8	1.3796 (19)	N3—S1	1.6260 (15)
C7—N3	1.4283 (19)	N3—H3N	0.8897
C8—H8	0.9300	O1—S1	1.4286 (11)
C9—C14	1.3884 (19)	O2—S1	1.4429 (11)
C9—C10	1.3974 (18)		
			
N1—C1—H1A	109.5	C10—C11—C12	120.42 (13)
N1—C1—H1B	109.5	C10—C11—H11	119.8
H1A—C1—H1B	109.5	C12—C11—H11	119.8
N1—C1—H1C	109.5	O3—C12—C13	124.39 (14)
H1A—C1—H1C	109.5	O3—C12—C11	115.54 (14)
H1B—C1—H1C	109.5	C13—C12—C11	120.08 (15)
N2—C2—C3	112.74 (13)	C14—C13—C12	119.65 (14)
N2—C2—Cl1	120.30 (11)	C14—C13—H13	120.2
C3—C2—Cl1	126.95 (12)	C12—C13—H13	120.2
C8—C3—C4	121.43 (13)	C13—C14—C9	120.33 (13)
C8—C3—C2	135.32 (14)	C13—C14—H14	119.8
C4—C3—C2	103.24 (13)	C9—C14—H14	119.8
N1—C4—C3	107.20 (13)	O3—C15—H15A	109.5
N1—C4—C5	131.85 (15)	O3—C15—H15B	109.5
C3—C4—C5	120.95 (14)	H15A—C15—H15B	109.5
C6—C5—C4	117.13 (15)	O3—C15—H15C	109.5
C6—C5—H5	121.4	H15A—C15—H15C	109.5
C4—C5—H5	121.4	H15B—C15—H15C	109.5
C5—C6—C7	122.58 (14)	N2—N1—C4	111.53 (13)
C5—C6—H6	118.7	N2—N1—C1	119.65 (15)
C7—C6—H6	118.7	C4—N1—C1	128.76 (15)
C8—C7—C6	120.47 (14)	C2—N2—N1	105.29 (12)
C8—C7—N3	123.47 (14)	C7—N3—S1	125.87 (10)
C6—C7—N3	115.98 (13)	C7—N3—H3N	116.4
C7—C8—C3	117.42 (14)	S1—N3—H3N	109.8
C7—C8—H8	121.3	C12—O3—C15	117.58 (15)
C3—C8—H8	121.3	O1—S1—O2	119.16 (7)
C14—C9—C10	120.00 (14)	O1—S1—N3	108.43 (7)
C14—C9—S1	119.80 (11)	O2—S1—N3	104.11 (7)
C10—C9—S1	120.20 (11)	O1—S1—C9	107.91 (7)
C11—C10—C9	119.45 (13)	O2—S1—C9	108.05 (7)
C11—C10—H10	120.3	N3—S1—C9	108.82 (7)
C9—C10—H10	120.3		

### Hydrogen-bond geometry (Å, º)

**Table:**

*D*—H···*A*	*D*—H	H···*A*	*D*···*A*	*D*—H···*A*
N3—H3*N*···O2^i^	0.89	2.04	2.9286 (17)	175
C5—H5···O3^ii^	0.93	2.56	3.426 (2)	156

Symmetry codes: (i) −*x*+2, −*y*+2, −*z*+1; (ii) *x*, *y*−1, *z*.
