# Supplementary figures and images for: Crystal structure of N-(3-chloro-1-methyl-1H-indazol-5-yl)-4-meth­oxy­benzene­sulfonamide
Source: Acta Crystallogr Sect E Struct Rep Online. 2014 Aug 9;70(Pt 9):o983–4. doi: 10.1107/S1600536814017747 (PMC4186104; doi:10.1107/S1600536814017747)

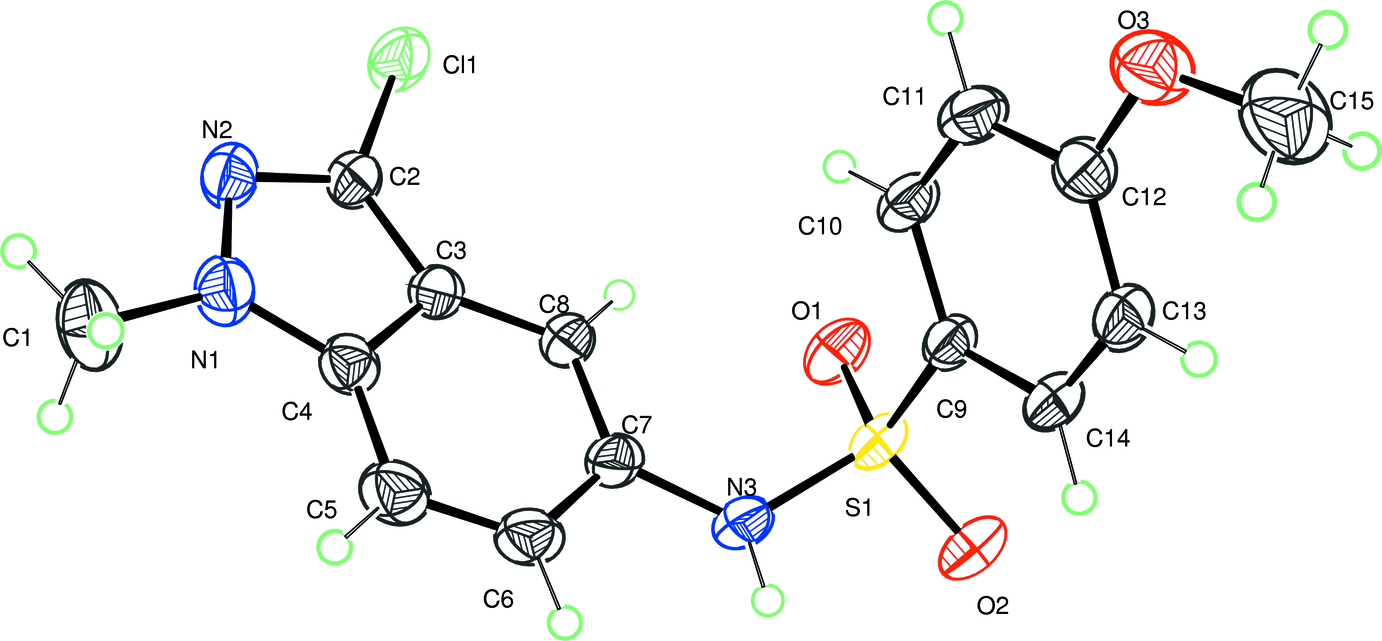

Supplement: Supplementary file 4 [file e-70-0o983-fig1.tif]

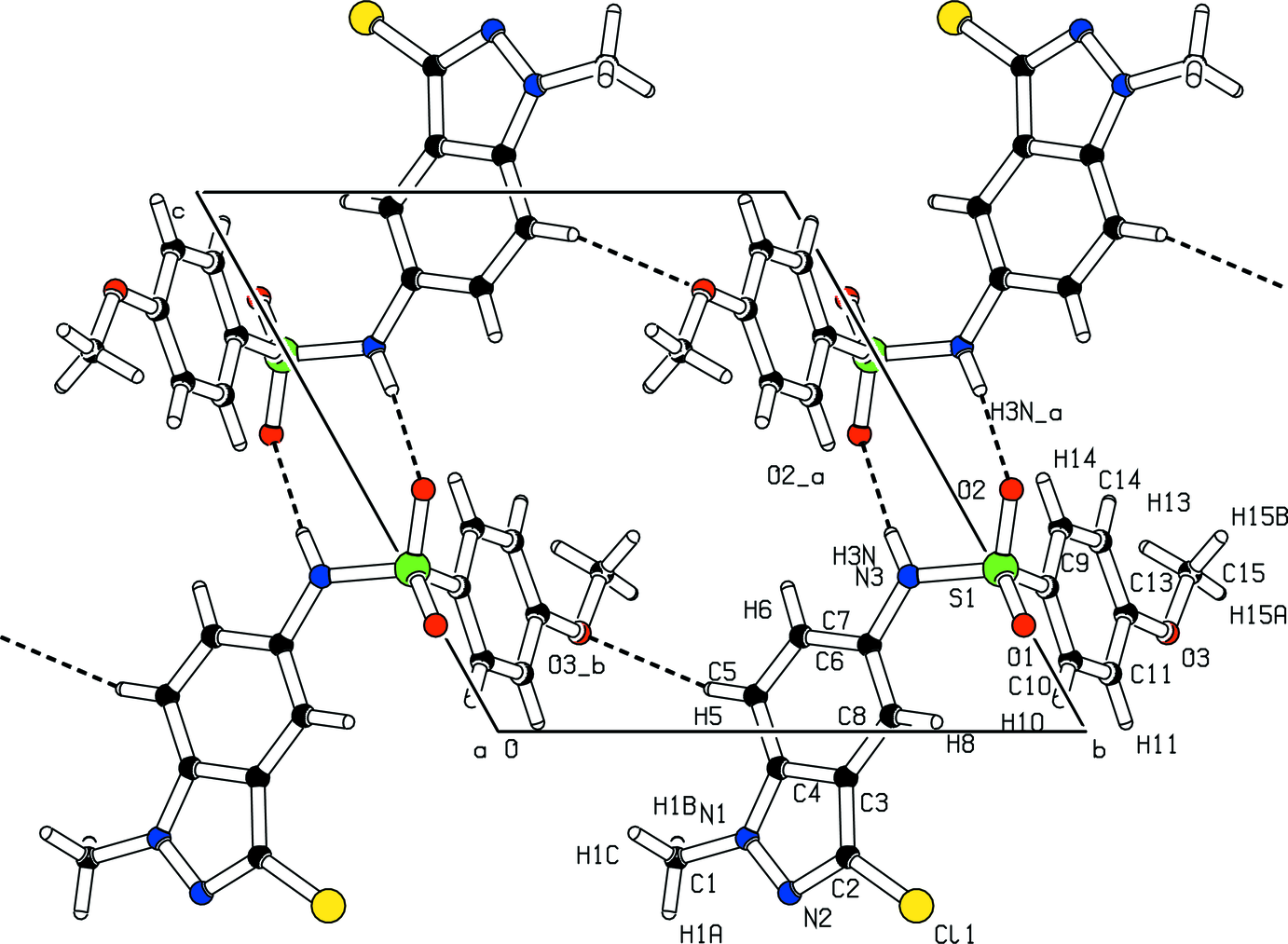

Supplement: Supplementary file 5 [file e-70-0o983-fig2.tif]
